# Response: Commentary: Neuromuscular and Muscle Metabolic Functions in MELAS Before and After Resistance Training: A Case Study

**DOI:** 10.3389/fphys.2020.00337

**Published:** 2020-04-15

**Authors:** Massimo Venturelli, Federico Ruzzante, Federica Villa, Doriana Rudi, Cantor Tarperi, Chiara Milanese, Valentina Cavedon, Cristina Fonte, Alessandro Picelli, Nicola Smania, Elisa Calabria, Spiros Skafidas, Stefania Fochi, Maria Grazia Romanelli, Gwenael Layec, Federico Schena

**Affiliations:** ^1^Department of Neurosciences, Biomedicine and Movement Sciences, University of Verona, Verona, Italy; ^2^Institute for Applied Life Sciences, University of Massachusetts Amherst, Amherst, MA, United States

**Keywords:** MELAS, resistance training, mitocondrial function, force, muscle mass, exercise

We thank Dr. Finsterer for his interest in our paper (Venturelli et al., 2019) and for pointing out some clinical factors that could improve the interpretation of the results in this case study. The main concerns expressed in the letter are related to the type of genetic defect and the rates of heteroplasmy of mtDNA (1), the degree of cardiac dysfunction (2), the nutritional status (3), the drugs regularly taken (4), and the cognitive abilities and cooperation of the subject (5) which based on the author's considerations, may need to be accounted for a detailed clinical diagnosis and treatment.

1) Despite the purpose of our investigation was to provide proof of concept that resistant training is feasible in a clinical population historically excluded from physical rehabilitation programs we have performed some lab tests to better elucidate Dr. Finsterer's queries by focusing on mitochondrial mutations and dys-function.

a) Determination of the most frequent (80%) mutation of mtDNA associated with MELAS [A3243G mutation in the mitochondrial tRNA^leu(UUR)^]. In order to determine this mtDNA mutation peripheral blood sample (10 mL) was collected in K2 EDTA-vacutainer. Genomic DNA was extracted from peripheral blood using salting-out procedure. Polymerase chain reaction amplification of the mtDNA portion coding for tRNA^leu(UUR)^ was performed using primers forward 5′-GCC TTC CCC CGT AAA TGA TA-3′ and reverse 5′-AGG TTG GCC ATG GGT ATG T-3′, previously described (Vivero et al., 2013). The presence of mtDNA A3243G mutation was verified by directed-DNA sequencing (BMR Genomics) and polymerase chain reaction-restriction fragment length polymorphism (PCR-RFLP) analysis. *ApaI* restriction enzyme (New England BioLabs) was used to digest PCR products and equal amounts of samples were then analyzed by electrophoresis through 7% polyacrylamide gels.

More than 80% of patients with MELAS carries the A3243G mutation in the mitochondrial tRNA^leu(UUR)^ gene (Goto et al., 1992). The presence of the A3243G mutation was investigated by direct DNA sequencing and by digestion with *ApaI* restriction enzyme of the amplified mitochondrial 161-bp PCR product. The presence of A3243G mutation generates an *ApaI* recognition site that yields two band of 87- and 74-bp fragments after restriction enzyme digestion. Both directed-DNA sequencing and PCR-RFLP analysis did not identify the A3243G mutation (Figures 1A,B).

**Figure 1:**
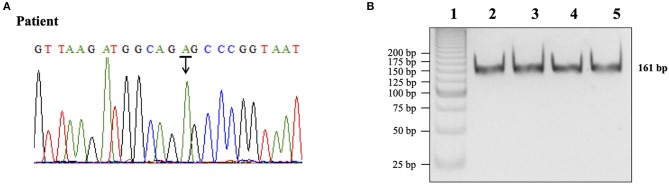
Determination of the A3243G mutation in the mtDNA portion coding for tRNA^leu(UUR).^ **(A)** DNA sequencing chromatogram for subject DNA product. The peak labeled with an arrow represented the presence of an adenine (A) in the 3243 region of the mtDNA gene. **(B)** Restriction enzyme analysis of PCR products. Lane 1,25 bp DNA ladder (Invitrogen); lane 2, normal control DNA product not digested; lane 3, normal control DNA product digested with *ApaI*; lane 4, patient DNA product not digested; lane 5, patient DNA product digested with *ApaI*.

b) To analyze mitochondrial function, we used peripheral blood mononucleated cells (PBMCs) from the patient and from three age and sex-matched controls. Blood was sampled from the antecubital vein, and PBMC were isolated by centrifugation on density gradients (Karabatsiakis et al., 2014). PBMCs were loaded in the O_2_K chambers of an Oxygraph-2k (Oroboros Instruments GmbH) for high resolution respirometry (HRR) and a SUIT (Substrate Uncoupler Inhibitor Titration) protocol for permeabilized cells was used for analysis (Doerrier et al., 2018). For the ROUTINE pyruvate (P) 5 mM and malate (M) 0.5 mM were added. Then cells were permeabilized with Digitonin (Dig) (10 mg/mL stock in DMSO) to allow specific substrates to enter the cells. The OXPHOS state was stimulated by adenine diphosphate (ADP) 1 mM to elicit oxidative phosphorylation. Glutamate (G) 10 mM was added to complete cI linked respiration. Succinate (S) was then added 10 mM to induce cII linked respiration (OXPHOS). Next carbonyl cyanide m-chlorophenyl hydrazine (CCCP) or uncoupler titrations (0.25 μM per step) were used to reach maximum uncoupled respiration, determining the maximal electron transfer capacity (ETS state). Finally, rotenone (Rot) 0.5 μM was added to inhibit cI, thus ETS sustained by cII activity was evaluated. Lastly Antimycin A 2.5 μM was used to inhibit cIII, and residual oxygen consumption (ROX) was measured. Data were analyzed with DatLab6 software (Oroboros Instruments GmbH) and expressed as flux control ratio (relative to ETS).

The qualitative data of flux control ratios (FCR) expressed relatively to maximal respiratory capacity of the uncoupled electron transfer system (ETS) show that mitochondria from the patient have a different pattern compared to controls. In particular ROUTINE and LEAK respiratory states oxygen consumption resulted significantly higher in mitochondria from the patient than controls (Figures 2A–C). Mitochondrial activity in the OXPHOS state sustained by complex I (pyruvate and malate) is increased by 23% in the patient compared to controls. This picture of mitochondrial dysfunction is accompanied by a decrease of mitochondrial efficiency compared to controls coupling efficiency, expressed as 1-L/E, suggesting that the quality of the link between electron transfer and ATP production is significantly lower in the patient mitochondria (Figure 2D). Higher levels in the ROUTINE and LEAK states, accompanied by lower efficiency in samples from the patient suggest a higher contribute of dissipative oxygen consumption, possibly due to an increased load of oxidative stress. These results are in line with the findings of a recent analysis of protein signature of metabolism and anti-oxidant response in clinically and genetically diagnosed patients with MELAS (Santacatterina et al., 2018). The authors show that the expression levels of some components of the electron transport chain associated to complex I (NADH9) are increased in MELAS patients. Similarly, the expression of ROS scavenging enzymes (SOD1 and SOD2) were changed (SOD1 increased, SOD2 decreased).

**Figure 2:**
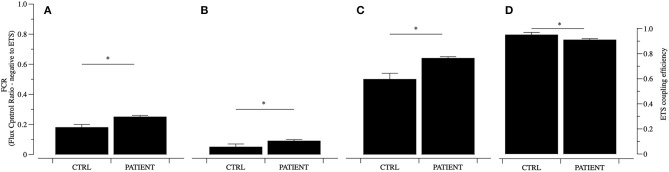
Mitochondrial function analyzed in permeabilized PBMCs. Mitochondrial function was analyzed using a substrate inhibitor titration (SUIT) protocol and data of Flux Control Ratio (FCR) relative to ETS are reported for controls (CTRL) and PATIENT. The ROUTINE **(A)**, LEAK **(B)**, OXPHOS cI (PM, **C**), respiratory states and ETS coupling efficiency (1-L/E, **D**) were analyzed. Data are represented as mean ± SD. *P < 0.05.

c) In order to determine blood lactate concentration [La^−^], arterialized blood samples (4 μL) were taken from the earlobe of the patient and 3 healthy controls. The [La^−^] was determined using a lactate analyzer (Labtrend LT14187; Bio Sensor Technoly GmbH, Berlin, Germany). The results showed that in the patient [La^−^] was 3.7 mmol/L wile in the controls was 0.7–1.0 mmol/L.

2) In his commentary, Dr. Finsterer, raised some concerns about the use of the pacemaker for the treatment of cardiac dysfunction in this patient, especially regarding potential systolic dysfunction, peripheral veins, and vascular abnormalities and exertional dyspnea as this would influence the “trainability” of the participant. The clinical reason of for the pacemaker implantation was a complete atrioventricular block. As to the influence of cardiovascular dysfunction on the training capacity of our participant, this is actually one of the key advantages of using resistance training rather than endurance exercise. Indeed, resistance training is less taxing on the cardiovascular system thus making this training method particularly suitable for patients with potential cardiac and vascular dysfunctions.
3) The nutritional status of the patient was actually monitored during the study. The amount of food consumed by the patient was ~1,800 kcal per day, with a consumption of carbohydrates corresponding to 55%, 20% of proteins and 25% fats.
4) The drugs regularly taken by the patient were only multivitamin supplements (vitamin B and C) and, above all, no changes were made to supplements during the 12 weeks of resistant training.
5) Dr. Finsterer has suggested that MELAS is characterized by fatigue, exercise intolerance and sleepiness, therefore is accessible only if the patient is highly motivated and willing to follow the instructions of the treating physicians. Certainly, the cognitive function of the patient (in our case study was worse than in healthy controls) but also mood and motivation are key factors for the success of any type of intervention, and even more so with exercise intervention.

Furthermore, we want to emphasize that the support and dedication of the main caregiver to this project was essential to achieve the high frequency rate. Only 5 times it was not possible for the main caregiver to follow the participant for the planned training and consequently they were missed. It is important to note that, this was the only reason for the 5 missing exercise sessions.

Finally, the numerous measurements (biopsies, MRS/I, oxidative stress, ATP production, mitochondrial membrane potential, apoptosis, and anti-oxidative capacity) suggested by Dr. Finsterer are all very interesting and would certainly shed light on the mechanisms underlying the training adaptations in this patient. In fact, in previous studies (Venturelli et al., 2014; Layec et al., 2018; Naro et al., 2019), we have performed such measurements in other populations and larger sample size. However, with such longitudinal project (3 months of training) and the unique challenges associated with the study of such a debilitating disease, we decided to favor its feasibility and to use non-invasive measurements.

In collusion, these results suggest that the patient investigated in this study was affected by MELAS-LIKE disease or a MELAS with different mutations rather than the A3243G. For this reason, in the original paper the term MELAS will be changed with MELAS-LIKE disease. Moreover, a limitation section will be amended to our paper (Venturelli et al., 2019) including these additional clinical data.

## Author Contributions

All authors listed have made a substantial, direct and intellectual contribution to the work, and approved it for publication.

### Conflict of Interest

The authors declare that the research was conducted in the absence of any commercial or financial relationships that could be construed as a potential conflict of interest.

## References

[B1] Doerrier C. Garcia-Souza L. F. Krumschnabel G. Wohlfarter Y. Meszaros A. T. Gnaiger E. (2018). High-resolution fluorespirometry and OXPHOS protocols for human cells, permeabilized fibers from small biopsies of muscle, and isolated mitochondria. Methods Mol. Biol. 1782, 31–70. 10.1007/978-1-4939-7831-1_3 29850993

[B2] Goto Y. Horai S. Matsuoka T. Koga Y. Nihei K. Kobayashi M.. (1992). Mitochondrial myopathy, encephalopathy, lactic acidosis, and stroke-like episodes (MELAS): a correlative study of the clinical features and mitochondrial DNA mutation. Neurology 42, 545–550. 10.1212/wnl.42.3.545 1549215

[B3] Karabatsiakis A. Bock C. Salinas-Manrique J. Kolassa S. Calzia E. Dietrich D. E.. (2014). Mitochondrial respiration in peripheral blood mononuclear cells correlates with depressive subsymptoms and severity of major depression. Transl Psychiatry 4:e397. 10.1038/tp.2014.44 26126180 PMC4080325

[B4] Layec G. Trinity J. D. Hart C. R. Le Fur Y. Zhao J. Reese V.. (2018). Impaired muscle efficiency but preserved peripheral hemodynamics and mitochondrial function with advancing age: evidence from exercise in the young, old, and oldest-old. J. Gerontol. A Biol. Sci. Med. Sci. 73, 1303–1312. 10.1093/gerona/gly050 29584857 PMC6132121

[B5] Naro F. Venturelli M. Monaco L. Toniolo L. Muti E. Milanese C.. (2019). Skeletal muscle fiber size and gene expression in the oldest-old with differing degrees of mobility. Front. Physiol. 10:313. 10.3389/fphys.2019.00313 30971947 PMC6443969

[B6] Santacatterina F. Torresano L. Nunez-Salgado A. Esparza-Molto P. B. Olive M. Gallardo E.. (2018). Different mitochondrial genetic defects exhibit the same protein signature of metabolism in skeletal muscle of PEO and MELAS patients: a role for oxidative stress. Free Radic. Biol. Med. 126, 235–248. 10.1016/j.freeradbiomed.2018.08.020 30138712

[B7] Venturelli M. Morgan G. R. Donato A. J. Reese V. Bottura R. Tarperi C. (2014). Cellular aging of skeletal muscle: telomeric and free radical evidence that physical inactivity is responsible and not age. Clin. Sci. 127, 415–421. 10.1042/CS20140051 24708050 PMC4757470

[B8] Venturelli M. Villa F. Ruzzante F. Tarperi C. Rudi D. Milanese C.. (2019). Neuromuscular and muscle metabolic functions in MELAS before and after resistance training: a case study. Front. Physiol. 10:503. 10.3389/fphys.2019.00503 31105594 PMC6498991

[B9] Vivero R. J. Ouyang X. Kim Y. G. Liu W. Du L. Yan D.. (2013). Audiologic and genetic features of the A3243G mtDNA mutation. Genet. Test. Mol. Biomark. 17, 383–389. 10.1089/gtmb.2012.0403 23477312 PMC3634140

